# NDUFA4L2 reduces mitochondrial respiration resulting in defective lysosomal trafficking in clear cell renal cell carcinoma

**DOI:** 10.1080/15384047.2023.2170669

**Published:** 2023-02-01

**Authors:** Jaclyn M. Kubala, Kristian B. Laursen, Ryan Schreiner, Ryan M. Williams, Johannes C. van der Mijn, Michael J. Crowley, Nigel P. Mongan, David M. Nanus, Daniel A. Heller, Lorraine J. Gudas

**Affiliations:** a Department of Pharmacology, Weill Cornell Medicine, New York, NY, USA; b Molecular Pharmacology Program, Memorial Sloan Kettering Cancer Center, New York, NY, USA; c Meyer Cancer Center, Weill Cornell Medicine, New York, NY, USA; d Division of Regenerative Medicine Research, Department of Medicine, Weill Cornell Medicine, New York, NY, USA; e Department of Biomedical Engineering, the City College of New York, New York, NY, USA; f Department of Physiology, Biophysics, and Systems Biology, Weill Cornell Medicine, New York, NY, USA; g Faculty of Medicine and Health Sciences, Center for Cancer Sciences, University of Nottingham, Sutton Bonington Campus, Loughborough, UK; h Division of Hematology and Medical Oncology, Department of Medicine, Weill Cornell Medicine, New York, NY, USA; i Department of Urology; New York Presbyterian Hospital, Weill Cornell Medicine, New York, NY, USA

**Keywords:** NDUFA4L2, ccRCC, lysosome, mitochondria, NPC2, expansion microscopy, kidney cancer, mass spectrometry, co-immunofluorescence, glycolysis, oxidative phosphorylation, mitochondrial respiration, Warburg effect, MISTR2, MISTR3, MIRCAF2, MIRCAF3, hypoxia, NDUFA4, HIF1a

## Abstract

In clear cell renal cell carcinoma (ccRCC), activation of hypoxic signaling induces NADH dehydrogenase (ubiquinone) 1 alpha subcomplex, 4-like 2 (NDUFA4L2) expression. Over 90% of ccRCCs exhibit overexpression of NDUFA4L2, which we previously showed contributes to ccRCC proliferation and survival. The function of NDUFA4L2 in ccRCC has not been fully elucidated. NDUFA4L2 was reported to reduce mitochondrial respiration via mitochondrial complex I inhibition. We found that NDUFA4L2 expression in human ccRCC cells increases the extracellular acidification rate, indicative of elevated glycolysis. Conversely, NDUFA4L2 expression in non-cancerous kidney epithelial cells decreases oxygen consumption rate while increasing extracellular acidification rate, suggesting that a Warburg-like effect is induced by NDUFA4L2 alone. We performed mass-spectrometry (MS)-based proteomics of NDUFA4L2 associated complexes. Comparing RCC4-P (parental) ccRCC cells with RCC4 in which NDUFA4L2 is knocked out by CRISPR-Cas9 (RCC4-KO-643), we identified 3,215 proteins enriched in the NDUFA4L2 immunoprecipitates. Among the top-ranking pathways were “Metabolic Reprogramming in Cancer” and “Glycolysis Activation in Cancer (Warburg Effect).” We also show that NDUFA4L2 enhances mitochondrial fragmentation, interacts with lysosomes, and increases mitochondrial-lysosomal associations, as assessed by high-resolution fluorescence microscopy and live cell imaging. We identified 161 lysosomal proteins, including Niemann-Pick Disease Type C Intracellular Cholesterol Transporters 1 and 2 (NPC1, NPC2), that are associated with NDUFA4L2 in RCC4-P cells. RCC4-P cells have larger and decreased numbers of lysosomes relative to RCC4 NDUFA4L2 knockout cells. These findings suggest that NDUFA4L2 regulates mitochondrial-lysosomal associations and potentially lysosomal size and abundance. Consequently, NDUFA4L2 may regulate not only mitochondrial, but also lysosomal functions in ccRCC.

## Introduction

Kidney cancer is one of the most common cancers, affecting over 300,000 people worldwide and causing 100,000 deaths per year,^1^ with clear cell renal cell carcinoma (ccRCC) accounting for approximately 80% of all kidney cancers.^2^ Clear cell RCCs can be highly aggressive, and although ccRCC-targeted therapies and immunotherapies have improved patient survival,^3^ the current treatment of advanced ccRCC is associated with a survival rate of only 8% for stage IV patients.^4,5^ Consequently, there remains a need for additional therapies for the prevention and treatment of advanced ccRCCs.

Clear cell RCC is generally caused by loss of function of the von Hippel-Lindau (VHL) tumor-suppressor gene.^6^ This results in constitutive activation of the transcription factor hypoxia-inducible factors−1α and −2α (HIF-1α and HIF-2α), leading to the transcriptional activation of hypoxia stimulated genes during normoxia.^7^ We previously demonstrated that NADH dehydrogenase [ubiquinone] 1 alpha subcomplex, 4-like 2 (NDUFA4L2), a HIF-1α target gene, is expressed at 40–60-fold higher levels in the majority (>90%) of ccRCC tumors relative to normal human kidneys.^8^ Additionally, ccRCC patients with the highest levels of NDUFA4L2 exhibit a lower survival rate.^8^ Our previous studies also suggest that NDUFA4L2 is critical for ccRCC cell proliferation, since knockdown of NDUFA4L2 in ccRCC cell lines results in decreased cell proliferation and decreased colony formation.^8^ NDUFA4L2 enhances the deposition of lipids and carbonic anhydrase 9 expression, a marker of ccRCC,^9^ in a murine ccRCC model.^10^ Therefore, NDUFA4L2 presents a novel molecular target for inhibition in ccRCC.

The function of NDUFA4L2 is not well understood. NDUFA4L2 is expressed in normal tissues primarily under hypoxic conditions, so it has not been studied extensively.^8^ NDUFA4L2 was shown to inhibit mitochondrial complex I activity in mouse embryonic fibroblasts (MEFs) and to be localized in mitochondria.^11^ We report here that NDUFA4L2 expression causes a Warburg-like shift from mitochondrial respiration to glycolysis, which is also reflected by increased numbers of fragmented mitochondria. Additionally, NDUFA4L2 expression increases the lysosomal diameter and decreases the number of lysosomes in RCC4 cells. Taken together, our data suggest that NDUFA4L2 functions to regulate both mitochondrial and lysosomal activities in ccRCC cells.

## Methods

### Cell culture

The RCC4 line (RCC4-P, P = Parental) is derived from a Von Hippel-Lindau (VHL) deficient primary human tumor (activated HIF1α signaling) and is used as a model for VHL-dependent mechanisms.^12–14^ The HK-2 cell line is an immortalized, non-tumorigenic human proximal tubule cell line with a functional, VHL-mediated repression of HIF1α. Both cell lines, from ATCC, were cultured in Dulbecco’s Modified Eagle’s Medium (DME) containing 10% Fetal Calf Serum (FCS, R&D Systems, S10250) with 5% CO_2_. Media was changed every 2–3 days, and cells were grown to 85% confluency in 15 cm plates. Once cells reached 85% confluency, cells were split using 0.25% Trypsin-EDTA 1X (Gibco, 25200–056). Cells were tested periodically for mycoplasma using PCR testing.

### Generation of cell lines

CRISPR/Cas9 constructs for generation of the NDUFA4L2 knockout (KO) line, RCC4-KO-643, were generated using the lentiCRISPR v2 vector (Addgene 52961) supplemented with a gRNA-targeting sequence (CTCAGGCGGTTCCAGGGCTC). Lentiviral transduction and puromycin selection of RCC4-P cells gave rise to the polyclonal knockout (KO) line RCC4-KO-643. Synthetic RNA (identical targeting sequence) was also used for generating NDUFA4L2 KO cells without antibiotic selection (Synthego). Polyclonal populations were genotyped by Next-Gen Amplicon Sequencing and analyzed in CRISPResso2.0. Single-cell dilutions were plated and individual clones were genotyped by PCR amplification, followed by TIDE analysis.^15^ This approach resulted in the generation of the monoclonal NDUFA4L2 KO line RCC4-Mc-2. The primers hNDUFA4L2(-)M (CCGGTCCTTCTTCAGCTTCTTA) and hNDUFA4L2(+)M (AGCCTCCGGGTGGAGCTTG) were used to generate a 326 bp amplicon spanning the gRNA targeting site. For more information on cell lines used and their corresponding experiments refer to Supplementary Table 1.

### Generation of HK-2-myc-FLAG-NDUFA4L2 cells

The *NDUFA4L2* coding sequence was amplified by PCR on cDNA derived from RCC4 cells. The myc-FLAG-NDUFA4L2 construct was generated by cloning PCR fragments into the empty pQCXIH vector from Clontech 631516 (PT3668). The cloning replaced the natural stop codon of NDUFA4L2 with a sequence coding for Myc- (EQKLISEEDL) and FLAG- (DYKDDDDK) epitopes. HK-2-Empty Vector (HK-2-EV) and HK-2-myc-FLAG-NDUFA4L2 (HK-2-F-NDU) cells were generated by hygromycin selection of HK-2 cells transduced with empty pQCXIH and myc-FLAG-NDUFA4L2-QCXIH expression vectors, respectively. Transgene expression was verified using western blotting for FLAG and NDUFA4L2 epitopes.

### Immunocytochemistry

We performed co-immunofluorescence (co-IF) on RCC4-Parental (RCC4-P) cells and RCC4-NDUFA4L2-KO (RCC4-KO-643) cells with NDUFA4L2 antibodies and different organelle/protein markers. Cells were seeded overnight and then treated with 50 µM MitoTracker Deep Red FM (ThermoFisher, M22426). Cells were then fixed with 4% paraformaldehyde (Electron Microscopy Sciences, 15710) and washed in phosphate buffered saline (PBS). Formaldehyde was quenched in 50 mM ammonium chloride for 20 minutes. Cells were permeabilized with either 0.1% Triton-X for 10 minutes or 0.075% saponin, and then blocked in 0.5% BSA (Roche, 10735086001) with 0.075% saponin for 1 h at room temperature. Primary antibody dilutions for NDUFA4L2 (Proteintech, 16480-1-AP) were prepared at 1:16,000 (high resolution and super-resolution microscopy) dilution in 0.075% saponin blocking solution and applied to all wells except the negative control wells. Cells were incubated in primary antibody dilutions for 1 h at room temperature and then at 4°C overnight. Cells were washed with blocking solution and secondary antibody solutions; anti-Rabbit (Alexa Fluor 488, Invitrogen 32790) and anti-Mouse (Alexa Fluor 594, Invitrogen 32744) were prepared at 1:500 (high resolution and super-resolution microscopy) in blocking solution. Cells were incubated in secondary solutions, washed first in blocking solution, and then in PBS. Cells were incubated with Hoechst Dye 33342 (ThermoFisher, H3570) nuclear stain dilution overnight at 4°C and then mounted in 2,2’-Thiodiethanol (Sigma Aldrich, 166782). Cell fluorescence was visualized and imaged within the next 1–3 hours. Primary antibodies used: LAMP1 (ThermoFisher, MA51812, 1:50), LAMP2 (Developmental Studies in Hybridoma Bank, H4B4, 1:50).

### Live cell imaging

Cells were seeded at 10,000 cells/well in 24-well plates (Cellvis, P24-1.5 H-N) overnight. Cells were cultured under non-starvation conditions (HBSS (Corning, 21–022-CV), 1% MEM nonessential amino acids (Corning, 25–025-Cl), 1% L-glutamine (Sigma, CAS#58-85-9), 1% serum), or starvation conditions (HBSS) for a total of 6 h. After 4 h, cells were treated with 50 µM MitoTracker Deep Red FM and 50 µM LysoTracker Green DND-26 (Invitrogen, Ref L7526). After 2 h with LysoTracker and MitoTracker, cells were imaged under normoxic conditions. After normoxic images were acquired, the cells were exposed to hypoxic conditions (1% to 5% oxygen) and imaged. For image acquisition, a widefield setup, including a 40X/1.4NA objective and Hamamatsu Flash4.0v2 sCMOS camera mounted on a confocal Zeiss Axio Observer Z1 was used with Zeiss Zen 2.6 acquisition software.

### Expansion microscopy

For expansion microscopy, cells were seeded at 10,000 cells/well on sterilized cover glass slips in 24-well plates (Cellvis, P24-1.5 H-N) and underwent co-IF as described above for super-resolution microscopy. After post-secondary PBS washes, cells were imaged using the LSM 880 microscope to acquire pre-expansion super-resolution microscopy images. The cells underwent anchoring treatment overnight in 0.1 mg/mL Acryloyl X-SE solution. The next day, the cells were washed twice in PBS for 15 minutes each and then treated with gelatin solution (10% TEMED, 10% ammonium persulfate (APS) for 1 h in 37°C incubator protected from light. Cells were digested overnight at room temperature with digestion buffer (50 mM Tris, 1 mM EDTA, 0.1% Triton X-100, 0.8 M guanidine HCl). The next day, the cells were washed in PBS for 5 min, and then again in PBS for 1 h. Cells were then imaged using the LSM 880 microscope to check for staining intensity. For weaker staining, cells were re-stained as described above for co-IF. After re-staining, the cells were expanded by washing 3–5 times in excess water (at least 10x the final gel volume) for 1 h each wash. Expanded cells were imaged using the LSM 880 laser-scanning confocal microscope.

### Western blotting

Western blotting was performed as previously described.^8^ Primary antibodies were used (1:1,000 in 5% milk): NDUFA4L2 (Abcam ab74138, rabbit polyclonal), NDUFA4L2 (Proteintech 66050-1-Ig, mouse monoclonal), FLAG (GenScript, A00187S, mouse monoclonal). Secondary antibodies: anti-Rabbit IgG (Jackson, 711–135-052, 1:10,000 diluted in 5% milk), anti-Mouse IgG (Jackson, 715–035-150, 1:10,000 diluted in 5% milk).

### Dynabead-antibody coupling

Dynabeads® M-270 Epoxy was coupled to NDUFA4L2 antibody (Abcam, ab74138) or Anti-Rabbit IgG (Cell Signaling, 2729) using the Life Technologies Dynabeads® Antibody Coupling Kit (14311D) as per manufacturer’s instructions.

### Immunoprecipitation (IP)

Cells were washed three times in cold PBS and harvested in radioimmunoprecipitation assay (RIPA) buffer. Cells were lysed on rockers for 30 min at 4°C, and then centrifuged at 13,000 g for 10 minutes at 4°C. Supernatant was collected, and protein was quantified. Protein lysate (2 mg) was incubated with NDUFA4L2 antibody-coupled Dynabeads for 12 hours at 4°C. Protein-antibody-bead complexes were then washed three times with a RIPA buffer and eluted in the final sample buffer at 95°C. Immunoprecipitation was performed on three separate biological repeats for each group.

### Mass spectrometry

After verifying that NDUFA4L2 was immunoprecipitated, we determined the proteins immunoprecipitated in RCC4-P versus RCC4-KO-643 cell extracts. We first loaded the immunoprecipitation samples (~1.5 mg each) onto a 12% Bis-Tris gel. Samples were electrophoresed into the gel, and the gel was fixed with the same Fixative Solution used with the Pierce Silver Stain Kit for Mass Spectrometry (ThermoFisher, 24600). The gel then underwent in-gel trypsin digestion, followed by peptide desalting and LC-MS/MS analysis for protein identification using an Easy-nLC 1200 coupled to an Orbitrap Fusion Lumos mass spectrometer. We used label-free spectral counting to quantitate protein abundance. MaxQuant software was used to process data and to search against the UniProt human protein database. The LC-MS/MS was performed on three biological replicates per group by Drs. Mengmeng Zhu and Guoan Zhang in the Weill Cornell Medicine LC-MS/MS Core.

### Proteomics analysis

Contaminating peptides (i.e. keratins and non-human proteins) were removed from the analysis. For proteomics analysis, intensity values of protein groups were used. RCC4-P IP samples were compared with either RCC4-KO-643 IP samples or IgG (control) IP samples. Each group contained 3 IP samples. Proteins under the following conditions were removed: Intensity = 0 in at least 5 of 6 replicates, or proteins with intensity recorded in only one replicate in both sets. Enrichment scores were calculated by computing the log2 ratio of RCC4-P:RCC4-KO-643 or RCC4-P:IgG. GO Cellular Component, Elsevier Pathway Collection, HumanCyc, GSEA MSigDB Hallmark Pathway, and GSEA Oncogenic Signature analyses were performed using the EnrichR database.^16–18^ The enrichment analysis from respective databases is presented in whole, without selective presentation. Heatmaps were generated using the Morpheus analysis software.^19^ Proteomic analysis was performed on three separate biological repeats for each sample group; the entire mass spectrometry experiment was performed three times starting with different frozen cell stocks.

### Statistical analysis and experimental robustness

All the experiments were performed at least three times (n = 3 or >3). Student *t* test was performed on at least three separate, independent experiments (n = 3 or >3) using the Graph Pad Prism 9 software. Proteomics data were analyzed with MaxQuant and Enrichr software.^16–18,20^ The p-values are calculated with Enrichr software. The p-value is computed from the Fisher's exact test, which is a proportion test that assumes a binomial distribution and independence for the probability of any gene belonging to any set.

## Results

### NDUFA4L2 elicits a glycolytic shift in both HK-2 and RCC4 ccRCC cells

**Figure 1.**
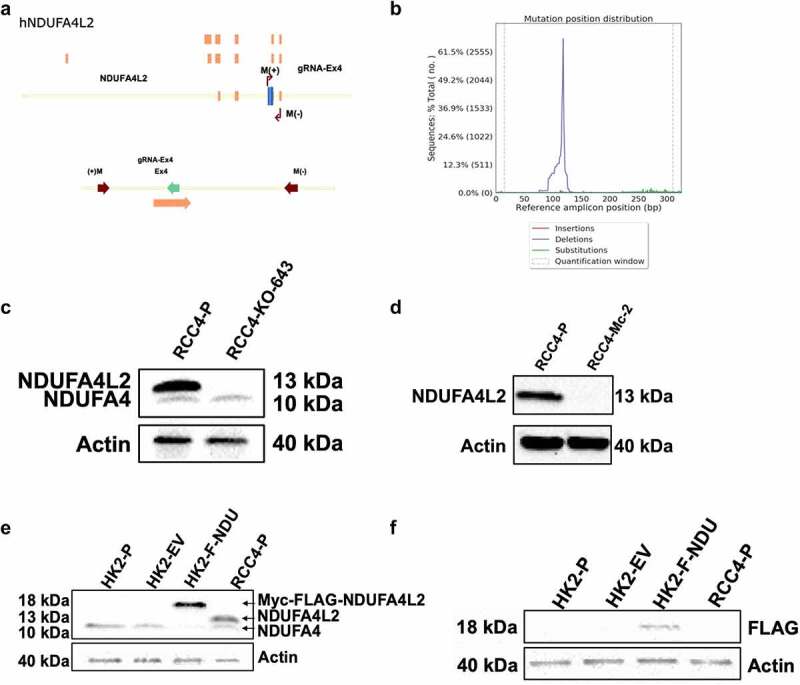
Caption: Generation of cell lines.

To assess the functions of NDUFA4L2 in ccRCC cells, we utilized a human ccRCC cell line RCC4 (RCC4-P) which expresses high levels of endogenous NDUFA4L2 in the presence of activated HIF-1α signaling.^8^ We first used CRISPR-Cas9 gene-editing technology to knock out (KO) NDUFA4L2 in RCC4-P (Figure 1a) and confirmed the NDUFA4L2 KO in the RCC4-KO-643 polyclonal cell line via amplicon next-generation sequencing (Figure 1b) and Western blotting (Figure 1c). Synthetic gRNAs were used to generate the single cell clonal NDUFA4L2 KO cell line RCC4-Mc-2, and we confirmed the complete KO using western blotting (Figure 1d). To model NDUFA4L2 in a cell line devoid of basal hypoxic signaling, we also generated an HK-2 cell line that expresses myc-FLAG-NDUFA4L2 (HK-2-F-NDU) and verified expression of both NDUFA4L2 and FLAG by immunoblotting (Figure 1e **&** figure 1f).

Figure 1 Alt Text: Genomic map of hNDJUFA4L2 with gRNA annotations (A). Graphical representation showing deletion frequency of NDUFA4L2 via Next-Generation Sequencing (B). Western blot that shows 13 kDa band for NDUFA4L2 present in RCC4-P cells but absent in RCC4-KO-643 cells (C). Western blot that shows 13 kDa band for NDUFA4L2 present in RCC4-P cells but absent in RCC4-Mc-2 cells (D). NDUFA4L2 Western blot showing 18 kDa band for Myc-FLAG-NDUFA4L2 present in HK2-F-NDU cells but absent in HK2-P and HK2-EV cells (E). FLAG Western blot showing 18 kDa band for Myc-FLAG-NDUFA4L2 present in HK2-F-NDU cells but absent in HK2-P, HK2-EV, and RCC4-P cells (F). Figure 1 legend: Genomic map of hNDUFA4L2 (A). CRISPR-Cas9 technology was used to knock out hNDUFA4L2 in RCC4 cells. Next-Generation Amplicon Sequencing of RCC4 cells containing NDUFA4L2 KO (RCC4-KO-643) cells showing deletion of NDUFA4L2 (B). Western blots of NDUFA4L2 and Actin in RCC4 parental (RCC4-P) cells and RCC4-KO-643 (C). Western blots of NDUFA4L2 and Actin in RCC4 parental (RCC4-P) cells and the monoclonal knockout line RCC4-Mc-2 (D). Samples were loaded at 25 μg of protein and electrophoresed on a 15% Tris-Glycine gel. Primary antibodies: NDUFA4L2 (Abcam ab74138, 1:1000 diluted in 5% milk), Actin (Millipore MAB1501, 1:10,000 diluted in 5% milk); Secondary antibodies: anti-Rabbit IgG (Jackson, 711–135-052, 1:10,000 diluted in 5% milk), anti-Mouse IgG (Jackson, 715–035-150, 1:10,000 diluted in 5% milk). The membrane was incubated with primary antibody dilutions overnight at 4°C. Secondary antibody incubation was performed for 2 hours at room temperature. All Western blots were performed in triplicate and repeated on 5 or more biological repeats. Western blots of NDUFA4L2 and Actin in HK-2 parental (HK-2-P), HK-2-Empty Vector (HK-2-EV), and HK-2-NDUFA4L2 (HK-2-F-NDU) (E). Western blots of FLAG and Actin in HK-2-P, HK-2-EV, HK-2-F-NDU, and RCC4-P cells (F). Samples were loaded (50 μg of protein/lane) and electrophoresed on a 15% Tris-Glycine gel. Primary antibodies: NDUFA4L2 (Abcam ab74138, 1:1000 diluted in 5% milk), FLAG (GenScript, A00187S, 1:1000 dilution in 5% milk), Actin (Millipore MAB1501, 1:10,000 diluted in 5% milk); Secondary antibodies: anti-Rabbit IgG (Jackson, 711–135-052, 1:10,000 diluted in 5% milk), anti-Mouse IgG (Jackson, 715–035-150, 1:10,000 diluted in 5% milk). NDUFA4 is a protein with an amino acid sequence related to that of NDUFA4L2; NDUFA4 is constitutively expressed in RCC4 cells under the cultural conditions used. NDUFA4L2 is the protein product of a separate gene. Membranes were incubated with primary antibody dilutions overnight at 4°C. Secondary antibody incubation was performed for 2 hours at room temperature. All Western blots were performed in triplicate and repeated on 5 or more biological replicates.

**Figure 2.**
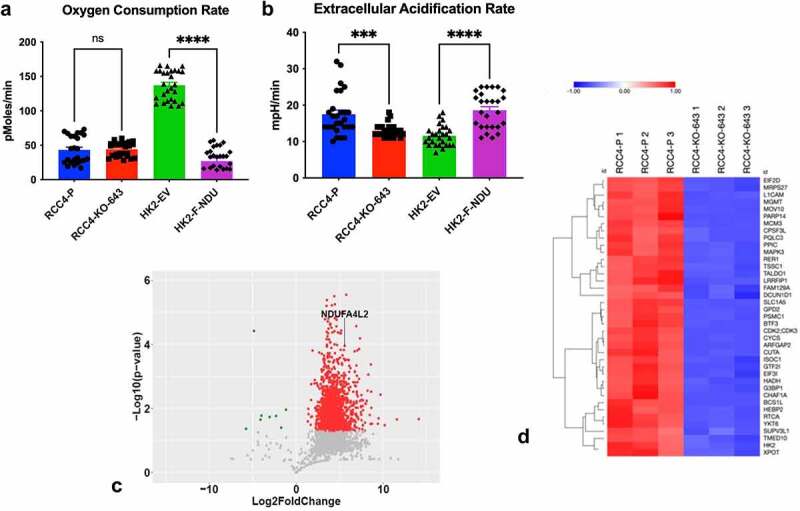
Caption: NDUFA4L2 decreases oxidative phosphorylation and increases glycolysis in renal cells.

NDUFA4L2 inhibits mitochondrial activity in MEFs.^11^ To determine if NDUFA4L2 acts as a metabolic switch in the kidney cells used in this study, causing a partial shift from oxidative phosphorylation to glycolysis, we measured the oxygen consumption rate, a measure of mitochondrial respiration (Figure 2a), and extracellular acidification rate, a measure of glycolysis (Figure 2b), using Seahorse XF technology in RCC4 and HK-2 parental and derivative cell lines. HK-2-F-NDU cells exhibited a 5-fold decrease (p < .0001) in oxidative phosphorylation compared to HK-2-EV (empty vector) control cells (Figure 2a). Additionally, NDUFA4L2 expression in HK-2-F-NDU cells increased glycolysis by 61-fold (p < .0001) compared to HK-2-EV cells (Figure 2b). We obtained similar results in the RCC4-KO-643 cells in which the KO of NDUFA4L2 resulted in a 35% decrease in glycolysis (p = .0004) (Figure 2b). Taken together, these results suggest that NDUFA4L2 expression can increase glycolysis in both RCC4 ccRCC cells and HK-2 cells.

Figure 2 Alt Text: Graph of oxygen consumption rate with vs. without NDUFA4L2 expression in cells. OCR in HK2-EV is significantly higher than OCR in HK2-F-NDU. RCC4-P and RCC4-KO-643 show no significant difference (A). Graph of extracellular acidification rate with vs. without NDUFA4L2 expression in cells. The ECAR in RCC4-P is significantly higher than ECAR in RCC4-KO-643. HK2-F-NDU ECAR is significantly higher than ECAR in HK2-EV cells (B). Volcano plot of NDUFA4L2 IP proteins in RCC4-P cells versus RCC4-KO-643 cells. Many proteins are IP-ed significantly in RCC4-P cells and fewer IP’ed significantly in RCC4-KO-643 cells (C). Heat map showing different top proteins that IP’ed significantly higher in RCC4-P cells versus RCC4-KO-643 cells. The heat map shows a clear difference, where higher IP’ed proteins in blue are in RCC4-P samples. These proteins are all lower (red) in RCC4-KO-643 cells (D).Figure 2 legend: Seahorse analysis of oxygen consumption rate (A) and extracellular acidification rate (B), an indicator of glycolysis, in RCC4-P versus RCC4-KO-643 cells and HK-2-EV versus HK-2-F-NDU cell lines. Cells were plated at 20,000 cells/well in a 96-well plate in 200 μL Seahorse media. After 16 hours, oxidative phosphorylation and glycolysis of cells were measured in triplicate. Assays were repeated for a total of three biological replicates. Volcano plot of proteins IP’ed in RCC4-P versus RCC4-KO-643 with cutoff at absolute Log_2_FC (C). Proteins that IP’ed at significantly higher levels in RCC4-P versus RCC4-KO-643 (p < 0.05) are highlighted in red. Proteins that IP’ed at significantly higher in RCC4-KO-643 versus RCC4-P (p < 0.05) are highlighted in green. All IP’s were performed in triplicate. Heat map of proteins that IP’ed at significantly greater level (p ≤ 0.0001) in RCC4-P versus RCC4-KO-643 cells (D).

### Mass spectrometry and proteomics analyses of RCC4 cells

To elucidate the actions of NDUFA4L2 is ccRCC, we then sought to identify proteins that bind to and/or are associated with NDUFA4L2 in RCC4 cells. We first performed an immunoprecipitation (IP) of NDUFA4L2 in the RCC4-P and RCC4-KO-643 cells (control). NDUFA4L2 immunoprecipitation was confirmed by performing IP-westerns in the HK-2-P (parental), HK-2-EV (empty vector), and HK-2-F-NDU cell lines and immunoblotting for both NDUFA4L2 and FLAG and detecting both NDUFA4L2 and FLAG proteins (**Supplementary Figure 1A, 1B**). The IP samples also immunoprecipitated a post-translationally modified NDUFA4L2 around 30 kDa in RCC4-P cells and 35 kDa in FLAG-NDUFA4L2 cells. After further investigation, we determined that some NDUFA4L2 is SUMOylated (**Supplementary Figure 1C, 1D**).

We next performed mass-spectrometry (MS)-based proteomics of NDUFA4L2 immunoprecipitates and identified 3,215 unique proteins (**Supplementary Figure 2A** and **Supplementary file 1**) that co-immunoprecipitated with NDUFA4L2. RCC4-P and RCC4-KO-643 IP samples were also compared to cells immunoprecipitated with a nonspecific isotype control (RCC4-IgG) to control for contaminants and other nonspecific proteins co-purifying with NDUFA4L2. A comparative analysis of RCC4-P versus RCC4-KO-643 cells (Figure 2c) and RCC4-P versus RCC4-IgG (**Supplementary Figure 2A**) using normalized spectral counts identified the differential expression patterns presented in the corresponding volcano plots (Figure 2c**, Supplementary Figure 2B**). We found that 1,889 proteins were present at 5-fold greater levels in the RCC4-P NDUFA4L2 interactome compared to RCC4-KO-643, versus 5 proteins that were similarly identified at 5-fold greater levels in the RCC4-KO-643 IP samples compared to RCC4-P (p < .05). Hierarchical cluster analysis of differentially bound proteins (p < .0001) showed that replicates clustered together, with a clear separation between the RCC4-P and -KO-643 IPs (Figure 2d).

### Pathway analysis for NDUFA4L2

**Figure 3.**
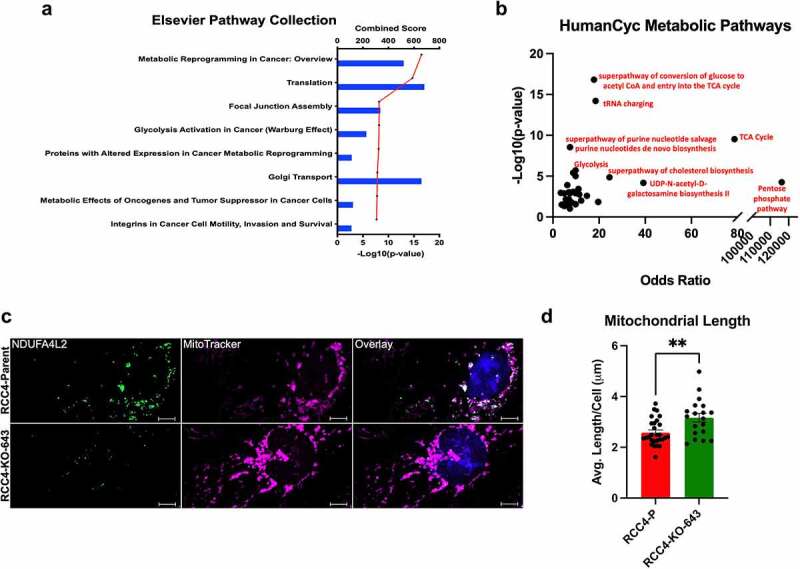
Caption: NDUFA4L2 alters mitochondrial length/fragmentation.

Previous studies have implicated NDUFA4L2 as a mitochondrial complex I inhibitor.^11^ Therefore, we first confirmed in our IP-MS studies that NDUFA4L2 is associated with the electron transport chain and mitochondrial complex I. We found that NDUFA4L2 is associated with 20 of the 45 subunits of mitochondrial complex I (log_2_^(Fold Change)^ ≥1, **Supplementary Table 2**). Additionally, NDUFA4L2 associated with other mitochondrial respiratory subunits, including complex II, III, IV, and ATP synthases (**Supplementary File 1**). We next performed a pathway analysis using the Elsevier Pathway Collection^16–18^ to identify the biological pathway components most strongly associated with NDUFA4L2 interacting proteins based on the fold changes (FC) between the RCC4-P IP and the RCC4-KO-643 IP (FC ≥ 2). We identified the top 20 pathways significantly enriched in the RCC4-P compared to the RCC4-KO-643 IP (Figure 3a) and found that among the highest-ranking pathways identified were “Metabolic Reprogramming in Cancer” and “Glycolysis Activation in Cancer (Warburg Effect)”. These analyses further support our data that NDUFA4L2 is involved in increasing the Warburg effect in ccRCC cells (Figure 3a).

Figure 3 Alt Text: Graphical representation of pathway analysis of proteins that IP’ed higher in RCC4-P cells versus RCC4-KO-643 cells. The top pathway identified is “Metabolic Reprogramming in Cancer: Overview,” and the fourth and fifth highest significant pathways identified are “Glycolysis Activation in Cancer (Warburg Effect)” and “Proteins with Altered Expression in Cancer Metabolic Reprogramming.” (A) Graphical representation of HumanCyc Metabolic Pathway analysis of proteins that IP’ed higher in RCC4-P cells versus RCC4-KO-643 cells. The top pathway identified is “Pentose Phosphate Pathway” (B). Co-IF images of RCC4-P versus RCC4-KO-643 cells. NDUFA4L2 images show green fluorescent staining in RCC4-P cells and little to no staining in RCC4-KO-643 cells. MitoTracker images show brighter, Longer mitochondrial staining in RCC4-KO-643 cells versus RCC4-P cells. Overlay images show GFP, MitoTracker, and DAPI overlay images (C). Graph showing mitochondrial length in RCC4-P versus RCC4-KO-643 cells. Average mitochondrial length is higher in RCC4-KO-643 cells (D). Figure 3 legend: Elsevier Pathway Collection analysis using Enrichr of proteins that immunoprecipitated higher in the presence of NDUFA4L2 in RCC4 cells (A). The top eight significant pathway hits are represented. Bars indicate the combined score of each pathway, which is the product of the – log_10_(p-value), computed with the Fisher's exact test, and the z-score computed by the correction to the test. A higher combined score indicates a higher chance of incidence. Each dot on the red line represents the statistical significance of each biological pathway as indicated by – log_10_(p-value). Pathways are ranked based on their significance from highest significance (top) to lowest significance (bottom). The HumanCyc pathway analysis of proteins enriched in RCC4-P versus RCC4-KO-643 using Enrichr (B). Bars indicate the combined score of each pathway. Each dot represents the statistical significance of each molecular function as indicated by – log_10_(p-value). Immunofluorescence for NDUFA4L2 (488) and MitoTracker (647) with Hoechst (DAPI) and fluorescence overlay in RCC4-P cells and RCC4-KO-643 cells (C). Primary antibodies: NDUFA4L2 (Proteintech 16480-1-AP, 1:50 dilution); Secondary antibodies: anti-Rabbit Alexa-fluor 488 (Invitrogen, 1:500 dilution). MitoTracker = 50 µM for 2 hours before fixation. Original magnification = 756X. Scale bar = 10 μm. Quantifications of mitochondrial length in RCC4-P versus RCC4-643-KO cells using Zen black desktop software (Zeiss) (D). All IF performed in triplicate.

After observing the significant enrichment of proteins involved in metabolic cancer reprogramming in cells expressing NDUFA4L2, we wanted to gain further insight into these metabolic pathways. We therefore performed a HumanCyc enrichment analysis^16–18^ to identify the specific metabolic pathways enriched in RCC4-P cells in which NDUFA4L2 is expressed (Figure 3b). The top identified pathway enriched in RCC4-P versus RCC4-KO-643 cells was the pentose phosphate pathway (PPP) (odds ratio = 116,242; p = .000056). Additionally, two of the top 10 significant pathways identified as enriched in RCC4-P involve purine metabolism (a super-pathway of purine nucleotide synthesis and purine nucleotides *de novo* biosynthesis). We previously reported that the knockdown of NDUFA4L2 caused a significant decrease in PPP intermediates and thereby reduced purine and pyrimidine synthesis.^8^ These data, along with our previously reported data, suggest that NDUFA4L2 causes glycolytic metabolites to be shunted to the PPP, increasing nucleic acid synthesis in RCC4 cells.

### NDUFA4L2 interacts with mitochondria and increases mitochondrial fragmentation

Although NDUFA4L2 was previously shown to inhibit mitochondrial respiration by inhibiting mitochondrial complex I,^11^ its subcellular location was not elucidated. We performed co-IF of NDUFA4L2 and MitoTracker using high-resolution microscopy of RCC4-P cells (Figure 3c). The RCC4-KO-643 cells served as a negative control. We discovered that NDUFA4L2 was associated with the outer mitochondria rather than located within the mitochondria (Figure 3c), as indicated by the co-localization of NDUFA4L2 fluorescence with the outermost part of the MitoTracker fluorescence.

To determine if NDUFA4L2 altered mitochondrial fragmentation in RCC4 cells, we measured mitochondrial fragmentation using Zen Blue desktop software (Zeiss) fiber length analysis. We found that mitochondria in RCC4-P cells are more fragmented than mitochondria in RCC4-KO-643 cells, and that RCC4-KO-643 cells exhibit longer, string-like mitochondrial networks (Figure 3c, 3d). RCC4-P cells exhibited an average of 0.58 µm (p = .0026) shorter mitochondrial lengths than RCC4-KO-643 cells, indicating increased fragmentation in RCC4-P cells (Figure 3d). These results suggest that knocking out NDUFA4L2 improves mitochondrial function, as defined by decreased mitochondrial fragmentation,^21^ in the RCC4-KO-643 cells. These data extend our previous findings that depletion of NDUFA4L2 results in improved mitochondrial morphology in RCC cells.^8^

### NDUFA4L2 associates with lysosomes

**Figure 4.**
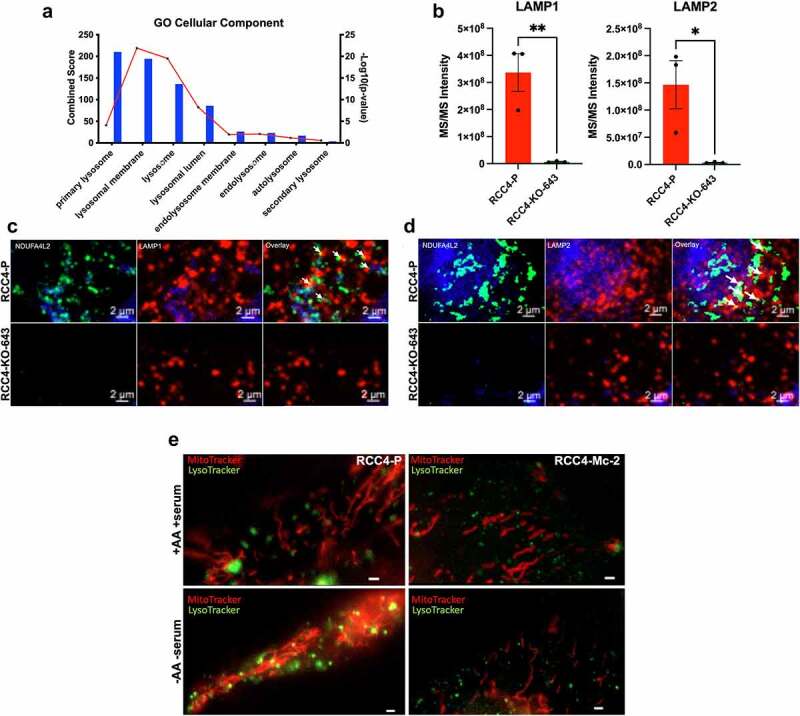
Caption: NDUFA4L2 associates with the lysosome.

When analyzing our MS data, we discovered that some proteins not localized in mitochondria co-immunoprecipitated with NDUFA4L2 in RCC4-P. Our cellular component analysis showed that many lysosomal proteins were enriched in the RCC4-P IPs compared to the RCC4-KO-643 IP’s (Figure 4a). ^16–18^ Using this cellular component bioinformatic analysis, we identified lysosomal associated membrane proteins 1 and 2 (LAMP1 and LAMP2), both well-established markers of the lysosome,^22^ in the NDUFA4L2 interactome. Specifically, LAMP1 and LAMP2 were 48-fold and 42-fold higher, respectively, in the RCC4-P compared to the RCC4-KO-643 immunoprecipitated samples (Figure 4b). In addition to these lysosomal markers, we identified a total of 161 lysosomal proteins that were associated with NDUFA4L2 (**Supplementary File 1**).

Figure 4 Alt Text: Graphical representation of GO Cellular Component analysis of proteins that IP’ed higher in RCC4-P cells versus RCC4-KO-643 cells that are known to localize and/or interact with the lysosome (A). Graph of MS/MS intensity comparing how strongly LAMP1 and LAMP2 IP’ed in RCC4-P versus RCC4-KO-643 cells. Both the LAMP1 and LAMP2 IP’ed were significantly higher in RCC4-P versus RCC4-KO-643 cells (B). Co-immunofluorescence in RCC4-P versus RCC4-KO-643 cells. RCC4-P cells show green fluorescence (NDUFA4L2) co-localizing with red fluorescence (LAMP1). RCC4-KO-643 cells show red fluorescence (LAMP1) (C). Co-immunofluorescence in RCC4-P versus RCC4-KO-643 cells. RCC4-P cells show green fluorescence (NDUFA4L2) co-localizing with red fluorescence (LAMP2). RCC4-KO-643 cells show red fluorescence (LAMP2) (D). Live cell imaging staining for mitochondrial (red) and lysosomes (green) in RCC4-P versus RCC4-Mc-2 cells. RCC4-P cells show more interactions between red and green fluorescent live stains compared to RCC4-Mc-2 cells (E). Figure 4 legend: Cellular component analysis using RStudio of lysosomal compartments identified in IP-MS (A). Bars indicate the number of genes identified in the mass spectrometry data that are known to be present in each respective cellular components. Bars indicate the combined score of each pathway, which is the product of the – log_10_(p-value), computed with the Fisher's exact test, and the z-score computed by the correction to the test, automatically calculated by the Enrichr software. A higher combined score indicates a higher chance of incidence. Each dot on the red line represents the statistical significance of each biological pathway as indicated by – log_10_(p-value). Pathways are ranked based on their combined score from highest (left) to lowest (right). MS/MS Intensity of LAMP1 and LAMP2 as observed by mass spectrometry proteomic analysis (B). Immunofluorescence for NDUFA4L2 (488) and LAMP1 (C) or LAMP2 (D) (594) with Hoechst (DAPI) and fluorescence overlay in RCC4-P cells. Primary antibodies: NDUFA4L2 (Proteintech 16480-1-AP, 1:16,000 dilution), LAMP1 (ThermoFisher MA51812, 1:50 dilution) LAMP2 (Developmental Studies in Hybridoma Bank, H4B4, 1:50 dilution);; Secondary antibodies: anti-Rabbit Alexa-fluor 488 (Invitrogen, 1:200 dilution), anti-Mouse Alexa-fluor 594 (Invitrogen, 1:200 dilution). Original magnification = 756X. Scale bar = 2 μm. Live cell video imaging of RCC4-P and RCC4-KO-Mc cells using MitoTracker (red) and LysoTracker (green) at 50 μM under nutrient-rich conditions or starvation conditions (E). For image acquisition, a widefield setup was used with Zeiss Zen 2.6 acquisition software. Live imaging was performed with a Zeiss Axio Observer Z.1 and a 20X/0.8NA or 40X/1.4NA objective. Recordings were acquired using a sCMOS with 6.5 µm^2^ pixels (Hamamatsu Flash4.0v2). Live experiments were performed within an incubation chamber controlled by the Zeiss Module S1 from Pecon. The cells were maintained at 37°C with 5% CO_2_ and high humidity. Original magnification = 400X. Scale bar = 10 μm.

We next performed high-resolution microscopy to visualize the association of NDUFA4L2 with lysosomes by co-IF (Figure 4c, 4d**, Supplementary Figure 3C**). We discovered that NDUFA4L2 was associated with both LAMP1 (Figure 4c) and LAMP2 (Figure 4d) in RCC4-P cells, as visualized by co-IF and defined by the co-localization of NDUFA4L2 fluorescence with both the LAMP1 and the LAMP2 fluorescence. This analysis indicates that NDUFA4L2, in addition to being a mitochondrial protein, is also associated with lysosomes, mainly on the periphery.

### NDUFA4L2 alters mitochondrial interactions with lysosomes

Since our proteomic analysis suggested that NDUFA4L2 interacts with proteins in both mitochondrial and lysosomal pathways, we aimed to determine if NDUFA4L2 altered interactions between mitochondria and lysosomes. We performed live imaging of RCC4-P and the monoclonal NDUFA4L2 KO cell line, RCC4-Mc-2, in which NDUFA4L2 is absent (Figure 4e) and visualized the mitochondria and lysosomes using MitoTracker and LysoTracker live staining fluorescent probes. We utilized the single-cell derived RCC4-Mc-2 line for these experiments to reduce the variability of single-cell imaging. Live imaging of the mitochondrial-lysosomal interactions showed that mitochondrial-lysosomal interactions are stronger in the RCC4-P cells, as defined by the number of lysosomes associated with mitochondria, compared to the RCC4-Mc-2 cells (Figure 4e**, Video Files 1–4)**.

Since nutrient starvation can alter lysosomal activity,^23^ we also performed live imaging under starvation conditions in which cells were deprived of amino acids and serum. We again observed that interactions between mitochondria and lysosomes were greater in the RCC4-P cells than the RCC4-Mc-2 cells (Figure 4e**, Video Files 1–4**). Additionally, we found that starvation conditions promoted association of mitochondria and lysosomes, as evidenced by spatial overlap of MitoTracker with LysoTracker, compared to nutrient-rich conditions, in the RCC4-P cells (Figure 4e**, Video Files 1–4**). In the RCC4-Mc-2 cells, we again observed decreased numbers of mitochondrial-lysosomal interactions under starvation conditions compared to those in RCC4-P cells. These findings suggest that NDUFA4L2 increases interactions between mitochondria and lysosomes, as defined by our live cell imaging of MitoTracker and LysoTracker dyes, and that mitochondrial-lysosomal associations are further enhanced under starvation conditions in RCC4-P cells.

### NDUFA4L2 elicits changes in lysosomal morphology and lysosomal trafficking

**Figure 5.**
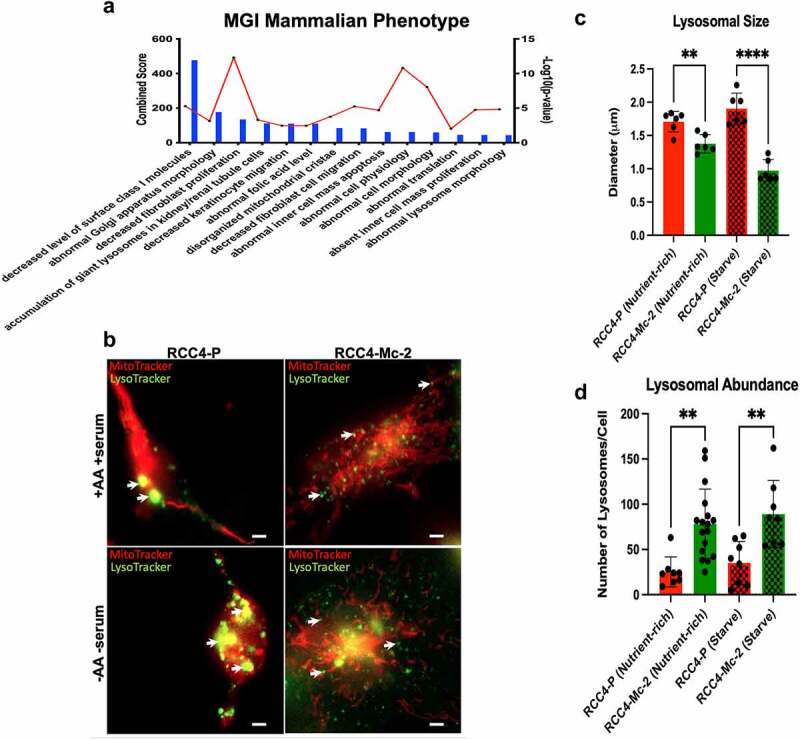
Caption: NDUFA4L2 alters lysosomal size, number, and organization.

The Warburg effect results in a more acidic tumor microenvironment as a result of increased lactic acid production.^24^ Previous studies investigating how the Warburg effect alters lysosomal trafficking in breast cancer cells found that cells were more invasive and metastatic under acidic conditions and formed larger lysosomes that were localized to the perinuclear region, altering lysosomal morphology and lysosomal trafficking.^25,26^ We next investigated if NDUFA4L2 altered lysosomal trafficking in RCC4 cells. MGI Mammalian Phenotype analysis^16–18^ of our IP-MS proteins showed that one of the highest-ranking phenotypes enriched in RCC4-P was “accumulation of giant lysosomes in kidney/renal tubule cells” (combined score = 112.14, p = .00049) (Figure 5a). We therefore assessed whether this predicted “accumulation of giant lysosomes” is observed in RCC4 cells in a NDUFA4L2-dependent manner in real time using a live imaging co-IF (Figure 5b). We found that RCC4-P cells exhibited an average of 0.33 µm (p = .0028) larger lysosomal diameters compared to RCC4-Mc-2 cells (1.7 ± 0.06 µm versus 1.38 ± 0.06 µm, respectively; Figure 5c). We also observed a 67% decrease in the numbers of lysosomes in RCC4-P compared to RCC4-Mc-2 cells (Figure 5d). These results support prior findings and are consistent with the data reported by Glunde *et al.*,^25^ in which a more acidic environment resulted in larger lysosomal diameter and altered lysosomal organization in cancer cells.

Figure 5 Alt Text: Graphical representation of MGI Mammalian Phenotype analysis of proteins that IP’ed higher in RCC4-P cells versus RCC4-KO-643 cells. One of the top phenotypes identified is “Accumulation of giant lysosomes in the kidney/renal tubule cells” (A). Live cell imaging staining for mitochondrial (red) and lysosomes (green) in RCC4-P versus RCC4-Mc-2 cells. RCC4-P cells show larger, fewer lysosomes compared to RCC4-Mc-2 cells. RCC4-Mc-2 cells show more abundant, smaller lysosomes. This difference was further enhanced in starvation conditions (B). Bar graph comparing lysosomal size of RCC4-P versus RCC4-Mc-2 cells with and without starvation conditions. RCC4-P cells have a much larger average lysosomal diameter compared to RCC4-Mc-2 cells. This difference was enhanced under starvation conditions (C). Bar graph comparing lysosomal abundance of RCC4-P versus RCC4-Mc-2 cells with and without starvation conditions. RCC4-P cells have a much lower number of lysosomes/cells compared to RCC4-Mc-2 cells. This difference was seen both in nutrient-rich conditions and under starvation conditions (D). Figure 5 legend: MGI Mammalian Phenotype analysis using Enrichr of proteins enriched with specific phenotypes NDUFA4L2 protein expression (A). Bars indicate the combined score of proteins identified in the mass spectrometry data that are known to be associated with the respective phenotype. Each dot of the red line represents the statistical significance of each biological pathway as indicated by – log_10_(p-value). Pathways are ranked based on their combined score from highest (left) to lowest (right). Live cell imaging of RCC4-P and RCC4-KO-Mc cells using MitoTracker (red) and LysoTracker (green) at 50 μM under nutrient-rich conditions or starvation conditions (B). For image acquisition, a widefield setup was used with Zeiss Zen 2.6 acquisition software. Live cell video imaging was performed with a Zeiss Axio Observer Z.1 and a 20X/0.8NA or 40X/1.4NA objective. Recordings were acquired using a sCMOS with 6.5 µm^2^ pixels (Hamamatsu Flash4.0v2). Live experiments were performed within an incubation chamber controlled by the Zeiss Module S1 from Pecon. The cells were maintained at 37°C with 5% CO_2_ and high humidity. Each condition consisted of two wells. For each well, five or more fields were acquired. All experiments were repeated in triplicate. Quantification of the lysosomal diameter (C) and lysosomal number (D) in RCC4-P versus RCC4-KO-Mc cells ± amino acids and serum using Zen Black desktop software (Zeiss). Original magnification = 400X. Scale bar = 10 μm.

Since we observed a change in mitochondrial-lysosomal interactions under starvation conditions (no serum or amino acids), we tested whether starvation conditions further enhanced the effects of NDUFA4L2 on lysosomal size and abundance in the RCC4 cells. As predicted, starvation conditions elicited a more pronounced effect on the lysosomal size, with lysosomal diameters over 2-fold larger in RCC4-P cells compared with RCC4-Mc-2 cells following 6 h of starvation conditions (p < .0001) (Figure 5c, 5d). The lysosomal diameters in RCC4-Mc-2 cells decreased further by an average of 0.40 µm under starvation conditions (0.9 ± 0.07 µm, p = .0010); in contrast, the lysosomal diameters in RCC4-P cells increased by 0.19 µm under starvation conditions (1.9 ± 0.1 µm) compared to those in RCC4-P cells under nutrient-rich conditions (Figure 5d). Taken together, these data suggest that NDUFA4L2 influences lysosomal size in ccRCC cells, and that these changes in lysosomal size, abundance, and interactions with mitochondria are further enhanced under starvation conditions.

### NDUFA4L2 associates with NPC1 and NPC2

**Figure 6.**
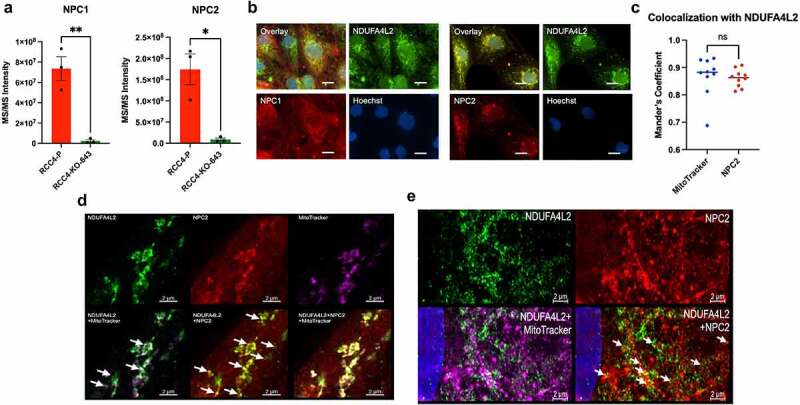
Caption: NPC-associated proteins immunoprecipitating with NDUFA4L2.

In addition to characteristic lysosomal markers, other proteins known to be localized in the lysosome immunoprecipitated with NDUFA4L2 (Figure 4a). Notably, Niemann-Pick disease type C1 and C2 (NPC1 and C2), which function together in regulating cholesterol transport and are known to localize in the lysosomes,^27,28^ immunoprecipitated at 29- and 19-fold greater extents, respectively, in RCC4-P cells compared with RCC4-KO-643 cells (Figure 6a).

Figure 6 Alt Text: Graph of MS/MS intensity comparing how strongly NPC1 and NPC2 IP’ed in RCC4-P versus RCC4-KO-643 cells. Both NPC1 and NPC2 IP’ed significantly higher in RCC4-P versus RCC4-KO-643 cells (A). Co-immunofluorescence in RCC4-P versus RCC4-KO-643 cells. RCC4-P cells show green fluorescence (NDUFA4L2) co-localizing with red fluorescence (NPC1 – left panel, NPC2 – right panel). RCC4-KO-643 cells show red fluorescence (NPC1 – left panel, NPC2 – right panel) (B). Mander’s coefficient of colocalization of MitoTracker and NDUFA4L2 (blue) and NPC2 and NDUFA4L2 (red) in RCC4-P cells. Both groups show similar colocalization coefficients (C). High resolution microscopy of co-immunufluorescent staining for NDUFA4L2 (green), NPC2 (red), and MitoTracker Deep Red. Overlay images show green fluorescence (NDUFA4L2) colocalizing with the MitoTracker Deep Red and red fluorescence (NPC2) (D). Super resolution microscopy of co-immunufluorescent staining for NDUFA4L2 (green), NPC2 (red), and MitoTracker Deep Red. Overlay images show green fluorescence (NDUFA4L2) is associated with the MitoTracker Deep Red and colocalizing with red fluorescence (NPC2) (E). Figure 6 legend: MS/MS Intensity of NPC1 and NPC2 as observed by mass spectrometry proteomic analysis (A). Immunofluorescence for NDUFA4L2 (488) and NPC1 or NPC2 (594) with Hoechst (DAPI) and fluorescence overlay in RCC4-P cells (B). Primary antibodies: NDUFA4L2 (Proteintech 16480-1-AP, 1:16,000 dilution), NPC2 (D3) (Santa Cruz sc-166449 AF594, 1:50 dilution); Secondary antibodies: anti-Rabbit Alexa-fluor 488 (Invitrogen, 1:500 dilution), anti-Mouse Alexa-fluor 594 (Invitrogen, 1:500 dilution). Magnification = 600X. Scale bar = 20 μm. Quantification of co-localization between NDUFA4L2 and either MitoTracker or NPC2 (C). Super-resolution microscopy of co-IF of NDUFA4L2 (488), MitoTracker (647), and NPC2 (D) (594) with Hoechst (DAPI) and fluorescence overlay in RCC4-P cells. MitoTracker = 50 µM for 2 hours before fixation. Primary antibodies: NDUFA4L2 (Proteintech 16480-1-AP, 1:50 dilution), NPC2 (D3) (Santa Cruz sc-166449 AF594, 1:50 dilution); Secondary antibodies: anti-Rabbit Alexa-fluor 488 (Invitrogen, 1:200 dilution), anti-Mouse Alexa-fluor 594 (Invitrogen, 1:500 dilution). Scale bar = 2 μm. Expansion microscopy of co-IF of NDUFA4L2 (488), MitoTracker (647), and NPC2 (594) with Hoechst (DAPI) and fluorescence overlay in RCC4-P cells (E) (2). MitoTracker = 50 µM for 2 hours before fixation. Primary antibodies: NDUFA4L2 (Proteintech 16480-1-AP, 1:50 dilution), LAMP2 (Developmental Studies in Hybridoma Bank, Catalog #H4B4, 1:50), NPC2 (H10) (Santa Cruz, sc-166321, 1:50); Secondary antibodies: anti-Rabbit Alexa-fluor 488 (Invitrogen, 1:500 dilution), anti-Mouse Alexa-fluor 594 (Invitrogen, 1:500 dilution). Scale bar = 2 μm. Super resolution and expansion microscopy experiments were performed in collaboration with the MSKCC Molecular Cytology Core Facility.

Singhal *et al*. previously reported 46 proteins that were differentially expressed by at least 2-fold or higher in patient-derived fibroblasts of healthy patients versus Niemann-Pick disease type C1 patients (NPC1 mutant).^29^ We found that 40 of these 46 proteins co-immunoprecipitated with NDUFA4L2 to a greater extent in RCC4-P cells versus RCC4-KO-643 cells (Table 1), suggesting that NDUFA4L2 is associated with NPC1 and further supporting the location of NDUFA4L2 in the early lysosomes. Thus, NDUFA4L2 may play a role in actions of the NPC1 protein.

**Table 1.** NPC-associated proteins immunoprecipitating with NDUFA4L2.

Gene Name	Log_2_(FC)	p-value
*PRKDC*	4.20	0.0287
*MYOF*	4.28	0.0225
*SSB*	4.63	0.0265
*NPEPPS*	3.81	0.0211
*G6PD*	3.96	0.0169
*RARS*	3.68	0.0174
*VCP*	4.04	0.0124
*EHD2*	4.67	0.0156
*STRAP*	5.26	0.0080
*ENO1*	3.23	0.0064
*CLIC1*	4.18	0.0060
*ETFA*	4.18	0.0190
*PA2G4*	4.23	0.0047
*DARS*	3.56	0.0131
*RUVBL2*	4.55	0.0408
*PSMD5*	4.12	0.0634
*PSAT1*	4.27	0.0141
*CAPZA2*	4.24	0.0398
*PCNA*	3.60	0.0003
*MMS19*	4.20	0.0188
*PSMD11*	3.72	0.0191
*MARS*	4.22	0.0238
*LAP3*	3.63	0.0289
*LAMP1*	5.60	0.0092
*HSPA1A*	4.48	0.0069
*ARPC4*	2.86	0.0477
*SARS*	4.09	0.0076
*SNX6*	4.01	0.0132
*RPL30*	3.48	0.0103
*ATP6V1 E1*	7.87	0.0949
*RPL14*	2.69	0.0672
*GLS*	4.46	0.0015
*PSMB4*	3.01	0.0011
*EIF3K*	4.70	0.0621
*RPS16*	3.28	0.0148
*PFN2*	3.21	0.0138
*AHNAK2*	3.61	0.0089
*CIAO1*	3.86	0.0260
*RPL23A*	2.79	0.1204
*FLNA*	6.72	0.1257

Proteins that IP’ed to a greater degree in the NDUFA4L2 interactome of RCC4-P compared to RCC4-KO-643. The respective genes/proteins have been shown to be altered in adipocytes upon mutation of NPC1, which associates with NDUFA4L2 (see Figure 6).

We next used co-IF to investigate the co-localization among NDUFA4L2, NPC1, and NPC2. We performed co-IF microscopy of NDUFA4L2 with NPC1 and NPC2. NDUFA4L2 co-localized with both NPC1 and NPC2 in RCC4-P cells (Figure 6b). Strikingly, we found that NDUFA4L2 co-localized with NPC2 with an association of 0.86 (Mander’s coefficient) in RCC4-P cells, which was similar to the association of NDUFA4L2 with mitochondria (Figure 6b, 6c). Taken together, these results indicate that NDUFA4L2 is also associated with these lysosomal NPC proteins.

There have been no previous reports of NDUFA4L2 interacting with NPC2 or other lysosomal proteins. Therefore, we further explored the association between NDUFA4L2 and NPC2 by expansion microscopy. We first visualized RCC4-P and RCC4-KO-643 cells fluorescently stained for NDUFA4L2, MitoTracker, and NPC2 using super-resolution microscopy (Figure 6d). The pre-expansion images suggest that NDUFA4L2 is associated with the lysosomal protein NPC2, as indicated by the spatial overlay of the NDUFA4L2 fluorescence and NPC2 fluorescence (Figure 6d). We next performed expansion microscopy, a technique in which cells are physically enlarged, resulting in a higher resolution image of these fluorescently stain cells.^30^ We observed that NDUFA4L2 was associated with NPC2, as indicated by the direct overlay of NDUFA4L2 and NPC2 fluorescence (Figure 6e). This spatial co-localization, combined with the association observed in the IP-MS data, supports the lysosomal function of NDUFA4L2 in RCC4 cells and suggests additional roles for NDUFA4L2 in clear cell renal cell carcinoma.

## Discussion

We previously reported that NDUFA4L2 is highly expressed at the mRNA and protein levels in ccRCC patients but is not expressed in normal human kidney tissue,^8^ making it a promising therapeutic target. Furthermore, elevated expression of NDUFA4L2 is associated with poorer survival in ccRCC patients.^8^ Published data on NDUFA4L2 functions are limited. Tello *et al*. reported that NDUFA4L2 is a HIF-1α target gene and that NDUFA4L2 inhibits mitochondrial complex I in HeLa cells and MEFs.^11^ NDUFA4L2 was also overexpressed in hepatocellular carcinoma^31^ and in glioblastoma, where elevated NDUFA4L2 levels also correlated with reduced patient survival.^32^

We and others have previously reported that HIF-1α enhances the Warburg effect in ccRCC, a common phenomenon observed in cancer in which cells exhibit a metabolic shift from mitochondrial respiration toward glycolysis.^33–35^ Here we demonstrate that NDUFA4L2 can increase glycolysis in RCC4 and HK-2 cells, suggesting that NDUFA4L2 can act independently of hypoxic signaling (Figure 2b). Additionally, RCC4-P cells exhibit increased mitochondrial fragmentation compared to RCC4-KO-643 cells, suggesting decreased mitochondrial activity in RCC4-P vs. RCC4 cells in which NDUFA4L2 is knocked out (Figure 3c**, 3D**). The decreased fragmentation of the mitochondria in the NDUFA4L2 KO, as demonstrated with fluorescence, correlates well with the improved mitochondrial morphology in NDUFA4L2 shRNA knockdown RCC4 cells, as demonstrated with electron microscopy, in a prior publication from our group.^10^ Furthermore, IP-MS analyses suggest that NDUFA4L2 is associated with increased PPP and nucleotide synthesis, which is indicative of a shift toward glycolysis and increased cell proliferation (Figure 3b).

Glykofridis *et al*. previously investigated how loss of FLCN-FNIP1/2 affects the proteome in human renal tubular epithelial cells.^36^ They generated FLCN knockout cells (FLCN^NEG^) using CRISPR/Cas9 technology in an immortalized, diploid renal proximal tubular cell line (RPTEC/TERT11). These KO cells were compared to the parental cell line (FLCN^POS^) using proteomic analysis. LC-MS/MS was utilized to identify a total of 5755 different proteins. Additionally, 914 differentially expressed proteins were identified that were expressed five-fold or higher in the FLCN^POS^ and FLCN^NEG^ cells.^36^ We aimed to utilize a similar approach in which we generated a NDUFA4L2 knockout line in a human renal cell carcinoma cell line (RCC4-KO-643) and then performed immunoprecipitations of RCC4-P and RCC4-KO-643 with an antibody to NDUFA4L2 to identify potential interacting proteins of NDUFA4L2 to gain further insight as to how NDUFA4L2 functions (Figure 2c, 2d).

We performed our IP and mass spectrometry experiments similarly to Takao *et al*., where they IP’ed MYB to identify 724 unique proteins associated with MYB in nuclear extracts of MV411 AML cells.^37^ After immunoprecipitating with an antibody to NDUFA4L2 in RCC4-P and RCC4-KO-643 cells, we performed mass spectrometry on these IP’ed samples. We identified a total of 1,894 differentially expressed proteins (p < .05) in the NDUFA4L2 interactome (Figure 2b**, 2C**).

Previous studies have suggested that NDUFA4L2 is located in the mitochondria in HeLa and MEFs.^11^ This, together with our seahorse analysis and the numbers of mitochondrial proteins in the IP-MS (**Supplementary Table 2**), led us to reason that NDUFA4L2 is located in the mitochondria in ccRCC cells. Interestingly, our co-IF experiments show that NDUFA4L2 is localized at the outer mitochondria (Figure 3c). Furthermore, our IP-MS and co-IF analyses suggest that NDUFA4L2 associates with lysosomes in ccRCC (Figure 4). This localization was further confirmed using expansion microscopy, which indicates that one of the proteins NDUFA4L2 co-localizes with is NPC2, a known lysosomal protein.^28^

Recent studies have investigated the importance of the lysosome and its cellular interactions in cancer.^23,38–42^ Interestingly, lysosomal interactions, particularly with the endoplasmic reticulum and mitochondria, can have major effects in increasing cancer progression and cancer signaling pathways.^38–40,42,43^ Previous studies have shown that contacts between the lysosome and mitochondria have a regulatory role in metabolism. These studies found that these mitochondrial-lysosomal contacts enable the efficient transfer of lysosomal metabolites into the mitochondrial matrix. This, in turn, fuels the tricarboxylic acid cycle.^38,44–47^ We found that RCC4-P cells exhibited a stronger association between mitochondria and lysosomes, as observed by our live cell imaging, compared to RCC4 cells that lack NDUFA4L2 (RCC4-Mc-2) (Figure 4e). This association was further enhanced in RCC4-P cells under starvation conditions (Figure 4e). These data demonstrate that NDUFA4L2 increases interactions between mitochondria and lysosomes in RCC4 cells, and that these interactions are further enhanced under starvation conditions.

Cancer cells typically exhibit a more acidic tumor microenvironment due to increased extracellular acidification, which is a result of increased glycolysis induced by the Warburg effect.^24–26,43,48,49^ We found that NDUFA4L2 expression resulted in an increase in glycolysis in both RCC4-P cells and HK-2-F-NDU cells (Figure 2b), therefore suggesting a lower pH environment when NDUFA4L2 is expressed. Glunde *et al*.^25^ found that more invasive, metastatic breast cancer cells exhibited larger and fewer lysosomes under more acidic pH conditions. We therefore reasoned that RCC4-P cells would exhibit increased lysosomal diameter and fewer lysosomes when NDUFA4L2 is expressed, which is what we observed (Figure 5). Previous work has shown that mitochondria-lysosomal contacts regulate lysosomal size via Rab7 hydrolysis.^50^ It is therefore possible that NDUFA4L2 is regulating lysosomal size in this manner, since NDUFA4L2 increased mitochondrial-lysosomal interactions, thereby resulting in increased lysosomal diameters (Figure 5).

Niemann Pick Type C proteins are involved in cholesterol trafficking and mitochondrial function. Defects in NPC1 and/or NPC2 result in lysosomal storage disease (LSD) and cholesterol accumulation within the lysosome.^51^ This cholesterol accumulation results in changes in lysosomal functions, morphology, and trafficking. Additionally, changes elicited by NPC defects directly affect other organelles, particularly the mitochondria. NPC2 is a soluble protein in the lumen of late endosomes and lysosomes. NDUFA4L2 may be part of a membrane contact region between lysosomes and mitochondria. Specifically, defects in NPC2 result in impaired mitochondrial function,^52^ similar to what we see when NDUFA4L2 is expressed. Here we report that, assessed by immunofluorescence, NDUFA4L2 co-localizes with both NPC1 and NPC2 (Figure 6), which are known lysosomal proteins, and that NDUFA4L2 alters mitochondrial-lysosomal interactions by promoting interactions between the mitochondria and the lysosome (Figure 4e). Furthermore, NDUFA4L2 expression alters lysosomal trafficking and morphology, as indicated by co-IF. We speculate that NDUFA4L2 could alter lysosomal trafficking and mitochondrial function in part through its interactions with NPC1 and NPC2. Large lysosomes are generally a consequence of defects in lysosomal hydrolysis and export, but defects in lysosomal size regulation can also lead to various diseases, including lysosomal storage disorders, such as Niemann-Pick disease, and cancer.^50^

In summary, the current study increases our understanding of NDUFA4L2, its role in RCC, and its potential role in several other malignancies in which NDUFA4L2 is overexpressed. Current and future studies are aimed to further delineate the molecular functions of NDUFA4L2 in ccRCC. Furthermore, based on our prior and current work, we plan to identify direct protein interactors in an effort to block the actions of NDUFA4L2 in ccRCC.

## Supplementary Material

Supplemental Material

## Data Availability

The authors confirm that the data supporting the findings of this study are available within the article [and/or] its supplementary materials.
