# Supplementary material for: NDUFA4L2 reduces mitochondrial respiration resulting in defective lysosomal trafficking in clear cell renal cell carcinoma
Source: Cancer Biol Ther. 2023 Feb 1;24(1):2170669. doi: 10.1080/15384047.2023.2170669 (PMC9897797; doi:10.1080/15384047.2023.2170669)
Supplement: Supplemental Material [file KCBT_A_2170669_SM7264.zip › 11232022_supplementary material for review with alt text _10132022_Jackie_MS LG 11_02_22.docx]

**Supplementary Information**

NDUFA4L2 reduces mitochondrial respiration resulting in defective lysosomal trafficking in clear cell renal cell carcinoma

Jaclyn M. Kubala^1,2,9^, Kristian B. Laursen^1^, Ryan Schreiner^3^, Ryan M. Williams^2,4^, Johannes C. van der Mijn^1^, Michael J. Crowley^5^, Nigel P. Mongan^1,6^, David M. Nanus^7,8,9^, Daniel A. Heller^1,2,5^, Lorraine J. Gudas^1,8,9^

^1^Department of Pharmacology; Weill Cornell Medicine, New York, NY 10065, USA

^2^Molecular Pharmacology Program, Memorial Sloan Kettering Cancer Center, New York, NY 10065, USA

^3^Division of Regenerative Medicine Research, Department of Medicine, Weill Cornell Medicine, New York, NY 10065, USA

^4^Department of Biomedical Engineering, The City College of New York, New York, NY 10031, USA

^5^Department of Physiology, Biophysics, and Systems Biology, Weill Cornell Medicine, New York, NY 10065, USA

^6^Faculty of Medicine and Health Sciences, Center for Cancer Sciences, University of Nottingham, Sutton Bonington Campus, Sutton Boninton, Leicestershire, UK

^7^Division of Hematology and Medical Oncology, Department of Medicine, Weill Cornell Medicine, New York, NY 10065, USA

^8^Department of Urology; New York Presbyterian Hospital, Weill Cornell Medicine, New York, NY 10065, USA

^9^Meyer Cancer Center, Weill Cornell Medicine, New York, NY 10065, USA

Correspondence:

Lorraine J. Gudas, PhD

Weill Cornell Medicine

Department of Pharmacology

1300 York Ave; New York, NY 10065

Telephone: 212-746-6250 ORCID #: 0000-0003-3115-4777

Email: [ljgudas@med.cornell.edu](mailto:ljgudas@med.cornell.edu)

**The authors declare no potential conflicts of interest.**

**Data available within the article or its supplementary materials:** The authors confirm that the data supporting the findings of this study are available within the article [and/or] its supplementary materials.

**Key words**: NDUFA4L2, ccRCC, lysosome, mitochondria, NPC2, expansion microscopy, kidney cancer, mass spectrometry, co-immunofluorescence, glycolysis, oxidative phosphorylation, mitochondrial

**Supplementary Information**

**METHODS**

***Immunocytochemistry***

In order to gain better insight to the function of NDUFA4L2, we visualized the location of NDUFA4L2 within ccRCC cells. We performed co-IF on RCC4-Parental (RCC4-P) cells and RCC4-NDUFA4L2-KO (RCC4-KO-643) cells with NDUFA4L2 antibodies and different organelle/protein markers. The immunofluorescence (IF) protocol was carried out as follows. Cells were seeded overnight at 5,000 cells/well in a 96-well glass bottom plate (Cellvis, P96-1.5H-N). The next day, cells were treated with 50 µM MitoTracker Deep Red FM (ThermoFisher, M22426) for 2 hours (no serum) and then fixed with 4% PFA (Electron Microscopy Sciences, 15710) for 20 minutes and washed in PBS for 10 minutes. Formaldehyde was quenched in 50 mM ammonium chloride for 20 minutes. Cells were permeabilized with either 0.1% Triton-X for 10 minutes or 0.075% saponin for 20 minutes at room temperature, and then blocked in 0.5% BSA (Roche, 10735086001) with 0.075% saponin for 1 hour at room temperature. Primary antibody dilutions for NDUFA4L2 (Proteintech, 16480-1-AP) were prepared at 1:16,000 (high resolution and super resolution microscopy) dilution in 0.075% saponin blocking solution and applied to all wells except the negative control wells (No Primary Antibody Control). Cells were incubated in primary antibody dilutions for 1 hour at room temperature and then at 4˚C overnight. Cells were washed with blocking solution six times for 5 minutes each. Secondary antibody dilutions for anti-Rabbit (Alexa Fluor 488, Invitrogen 32790) and anti-Mouse (Alexa Fluor 594, Invitrogen 32744) were prepared at 1:500 (high resolution and super resolution microscopy) in blocking solution and cells were incubated in secondary solutions for 1 hour and room temperature. Cells were again washed in blocking solution 6 times for 5 minutes and then washed twice in 1X PBS. Cells were incubated with Hoechst Dye 33258 (Calbiochem, 382061) nuclear stain dilution (10 µl Hoechst Dye in 30 mL 1X PBS) overnight at 4˚C overnight and then fixed in 2,2’-Thiodiethanol (TDE) (Sigma Aldrich, 166782). Cell fluorescence was visualized and imaged within the next 1-3 hours. For standard microscopy, images were acquired using the Nikon Eclipse TE2000-E microscope and NIH Elements AR 3.2 software using the GFP, TXRED, FARRED, and DAPI channels. For high resolution microscopy, imaging was performed on a Zeiss Cell Observer SD confocal with a Yokogawa CSU-X1 spinning disk using Plan-Apochromat 100X/1.4, 63X/1.4, and 20X/0.8 objectives paired with a 1.2X adapter to a Photometrics Evolve 512 EMCCD camera. The laser lines utilized are 405 nm, 488 nm, 561 nm, and 639 nm. For super resolution microscopy, images were acquired with a Zeiss LSM 880 area scanning microscope. Primary antibodies used: LAMP1 (ThermoFisher MA51812, 1:50) , LAMP2 (Developmental Studies in Hybridoma Bank, Catalog #H4B4, 1:50).

***Live Cell Imaging***

Live imaging was performed with a Zeiss Axio Observer Z.1 and a 20X/0.8NA or 40X/1.4NA objective. Recordings were acquired using a sCMOS with 6.5µm^2^ pixels (Hamamatsu Flash4.0v2). Live experiments were performed within an incubation chamber controlled by Zeiss Module S1 from Pecon. The cells were maintained at 37˚C with 5% CO_2_ and high humidity and varying oxygen concentrations from normoxia to 1% or 5% oxygen for hypoxic conditions.

***Silver Staining***

We first fixed the gel two times in Fixing Solution (30% ethanol, 10% acetic acid) for 15 minutes each at room temperature. We then washed the gel in 10% Ethanol Wash for 5 minutes each. The gel was then washed in ultrapure water two times for five minutes each. Just before use, we prepared the Sensitizer Working Solution. We then incubated the gel in the Sensitizer Working Solution for exactly one minute, and then washed the gel in ultrapure water two times for one minute each. We prepared the Silver Stain Solution and immediately added it to the gel and incubated for 5 minutes. While the gel was incubating, we prepared the Developer Working Solution (1 part Silver Stain Enhancer with 100 parts Silver Stain Developer). We then quickly washed the gel two times for 20 seconds each and then incubated the gel in Developer Working Solution until the protein bands appeared. To stop the reaction, we decanted the Developer Working Solution and added the Stop Solution (5% acetic acid) to the gel. After a 10 minute incubation, the gel was imaged using the Bio-Rad Imager (Bio-Rad). All solutions were provided in the kit.

**TABLES**

**SUPPLEMENTARY TABLE 1**

| **Cell line** | **VHL** | **NDUFA4L2** | **Figures** | **RRID** | **Experiments** |
| --- | --- | --- | --- | --- | --- |
| RCC4-P | Neg | Pos | Fig.1,2,3,4,5,  6D  6E | CVCL_0498 | Western blots  Super Resolution  Expansion Micro |
| RCC4-KO-643 | Neg | Neg | Fig.1BC  2AB  2CD  3C  4CD  6B | NA | Western blots  OCR/ECAR  IP/MS  coIF High Res  coIF Microscopy coIF Microscopy |
| RCC4-Mc-2 | Neg | Neg | Fig.1D,  4E,5B | NA | Western blots  Live Imaging |
| HK-2 | Neg | Neg | Fig.1G | CVCL_0302 | Western blots |
| HK-2-EV | Neg | Neg | Fig.1G  2  S1 | NA | Western blots  OCR/ECAR  IP |
| HK-2-F-NDU | Neg | Pos | Fig.1G  2  S1 | NA | Western blots  OCR/ECAR  IP |
| HEK293-F-NDU |  |  | Fig.S1B |  | Western blots |
| HEPG2 |  |  | Fig.S1D |  | Western blots |

**SUPPLEMENTARY TABLE 2**

| **Complex I subunits** | **Subunit type** | **Log_2_(FC)** | **p-value** |
| --- | --- | --- | --- |
| *NDUFS1* | core subunit | 3.97 | 0.0611 |
| *NDUFS3* | core subunit | 3.87 | 0.0242 |
| *NDUFS2* | core subunit | 3.41 | 0.0400 |
| *NDUFA4L2* | accessory subunit | 5.56 | 0.0001 |
| *NDUFA10* | accessory subunit | 3.47 | 0.0490 |
| *NDUFA11* | accessory subunit | 3.49 | 0.0496 |
| *NDUFV2* | core subunit | 5.19 | 0.0066 |
| *NDUFV1* | core subunit | 3.58 | 0.0798 |
| *MT-ND4* | core subunit | 3.79 | 0.0532 |
| *NDUFB4* | accessory subunit | 3.19 | 0.1182 |
| *NDUFB6* | accessory subunit | 8.63 | 0.0094 |
| *NDUFB7* | accessory subunit | 4.43 | 0.0033 |
| *NDUFB9* | accessory subunit | 3.35 | 0.1727 |
| *NDUFB10* | accessory subunit | 3.50 | 0.0598 |
| *NDUFC2* | accessory subunit | 3.30 | 0.1547 |
| *NDUFA5* | accessory subunit | 5.43 | 0.0030 |
| *NDUFS7* | core subunit | 3.88 | 0.1390 |
| *NDUFA9* | accessory subunit | 4.82 | 0.0406 |
| *NDUFA13* | accessory subunit | 2.21 | 0.2243 |
| *ACAD9* | assembly | 3.34 | 0.0253 |
| *NDUFAF4* | assembly | 4.08 | 0.0524 |

*Supplementary Table 1. Cell Lines.*

Cell lines and their expression of VHL and NDUFA4L2 with the corresponding experiments in which they were utilized.

*Supplementary Table 2. Mitochondrial complex I subunits immunoprecipitated to a greater degree in RCC4-P versus RCC4-KO-643.*

Mitochondrial complex I subunits immunoprecipitated with NDUFA4L2 after mass spectrometry analysis of IP samples. All identified subunits IP’ed at 2-fold or greater levels in the RCC4-P vs. RCC4-KO-643 cells. Subunits that IP’ed lower than 2-fold were excluded from the list.

**FIGURES**

**SUPPLEMENTARY FIGURE 1**

**B**

**A**

**SUPPLEMENTARY FIGURE 2**

**Supplementary Figure 1 Caption: Immunoprecipitation of NDUFA4L2 in HK-2 cells.**

**Supplementary Figure 1 Alt Text:**

NDUFA4L2 Western blot showing bands for Myc-FLAG-NDUFA4L2 present in HK2-F-NDU cell lysate and a more intense band for Myc-FLAG-NDUFA4L2 IP samples (A).

FLAG Western blot showing band for Myc-FLAG-NDUFA4L2 present in HK2-F-NDU cell lysate and a more intense band for Myc-FLAG-NDUFA4L2 IP samples (B).

SUMO 2/3/4 Western blot showing band for SUMOylated NDUFA4L2 present in HK2-F-NDU and RCC4-P IP samples (C).

NDUFA4L2 Western blot showing bands for Myc-FLAG-NDUFA4L2 present in RCC4-P sequential IP samples (D).

**Supplementary Figure 1 Legend:**

Western blot for NDUFA4L2 in HK2-EV and HK2-F-NDU cell lines after immunoprecipitation of NDUFA4L2 (A). Input samples = 25 μg; IP sample = 1/7 (5 μL) of total IP. FLAG Western blot HK2 cell lines after immunoprecipitation of NDUFA4L2 with 293T-F-NDU lysate as a positive control for FLAG (B). Samples were loaded and electrophoresed on a 15% Tris-Glycine gel. Primary antibodies: NDUFA4L2 (Abcam ab74138, 1:1000 diluted in 5% milk), FLAG (GenScript, cat#: A00187S, 1:1000 dilution in 5% milk), Actin (Millipore MAB1501, 1:1000 diluted in 5% milk); Secondary antibodies: anti-Rabbit IgG (Jackson, 711-135-052, 1:10,000 diluted in 5% milk), anti-Mouse IgG (Jackson, 715-035-150, 1:10,000 diluted in 5% milk). Membrane was incubated with primary antibody dilutions overnight at 4˚C. Secondary antibody incubation was performed for 2 hours at room temperature. Identification of SUMOylated NDUFA4L2. Western blot analysis of SUMO 2/3/4 protein expression in IP samples (C). HepG2 lysate was used as a positive control for SUMO expression. Western blot analysis of NDUFA4L2 protein expression in sequential IP (Seq IP) of NDUFA4L2 and SUMO 2/3/4 in RCC4 cells (D). Samples were loaded at 50 μg of protein (input) or ¼ IP lysate (25 μL) and electrophoresed on a 15% Tris-Glycine gel. Primary antibodies: NDUFA4L2 (Proteintech 66050-1-Ig, 1:1000 diluted in 5% milk), SUMO 2/3/4 (Santa Cruz, cat. # sc-393144, 1:1000 dilution in 5% milk), Actin (Millipore MAB1501, 1:1000 diluted in 5% milk); Secondary antibodies: anti-Rabbit IgG (Jackson, 711-135-052, 1:10,000 diluted in 5% milk), anti-Mouse IgG (Jackson, 715-035-150, 1:10,000 diluted in 5% milk). Membrane was incubated with primary antibody dilutions overnight at 4˚C. Secondary antibody incubation was performed for 2 hours at room temperature. All Western blots were performed in triplicate.

**Supplementary Figure 2 Caption:. Proteomic analysis of NDUFA4L2 immunoprecipitation samples.**

**Supplementary Figure 2 Alt Text:**

Heat map of all proteins that IP’ed significantly in RCC4-P versus RCC4-KO-643 cells for NDUFA4L2 IP. Proteins that IP’ed significantly higher are indicated in red whle proteins that IP’ed significantly lower are indicated in blue (A).

Volcano plot of NDUFA4L2 IP proteins versus IgG IP proteins in RCC4-P cells. Many proteins IP’ed significantly with NDUFA4L2 IP and few IP’ed significantly in IgG (B).

**Supplementary Figure 2 Legend:**

Volcano plot of NDUFA4L2/IgG IP in RCC4-P cells (A). Proteins that IP’ed significantly higher in RCC4-P versus IgG (p$<$0.05) are highlighted in red. Proteins that IP’ed significantly higher in IgG versus RCC4-P (p$<$0.05) are highlighted in green. All IP’s were performed in triplicate. Heat map of all proteins that IP’ed in RCC4-P versus RCC4-KO-643 cells (B).
