# Supplementary figures and images for: NDUFA4L2 reduces mitochondrial respiration resulting in defective lysosomal trafficking in clear cell renal cell carcinoma
Source: Cancer Biol Ther. 2023 Feb 1;24(1):2170669. doi: 10.1080/15384047.2023.2170669 (PMC9897797; doi:10.1080/15384047.2023.2170669)

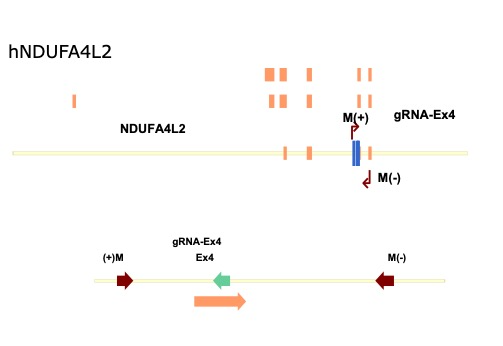

Supplement: Supplemental Material [file KCBT_A_2170669_SM7264.zip › Suppl Figure 1A.tiff]

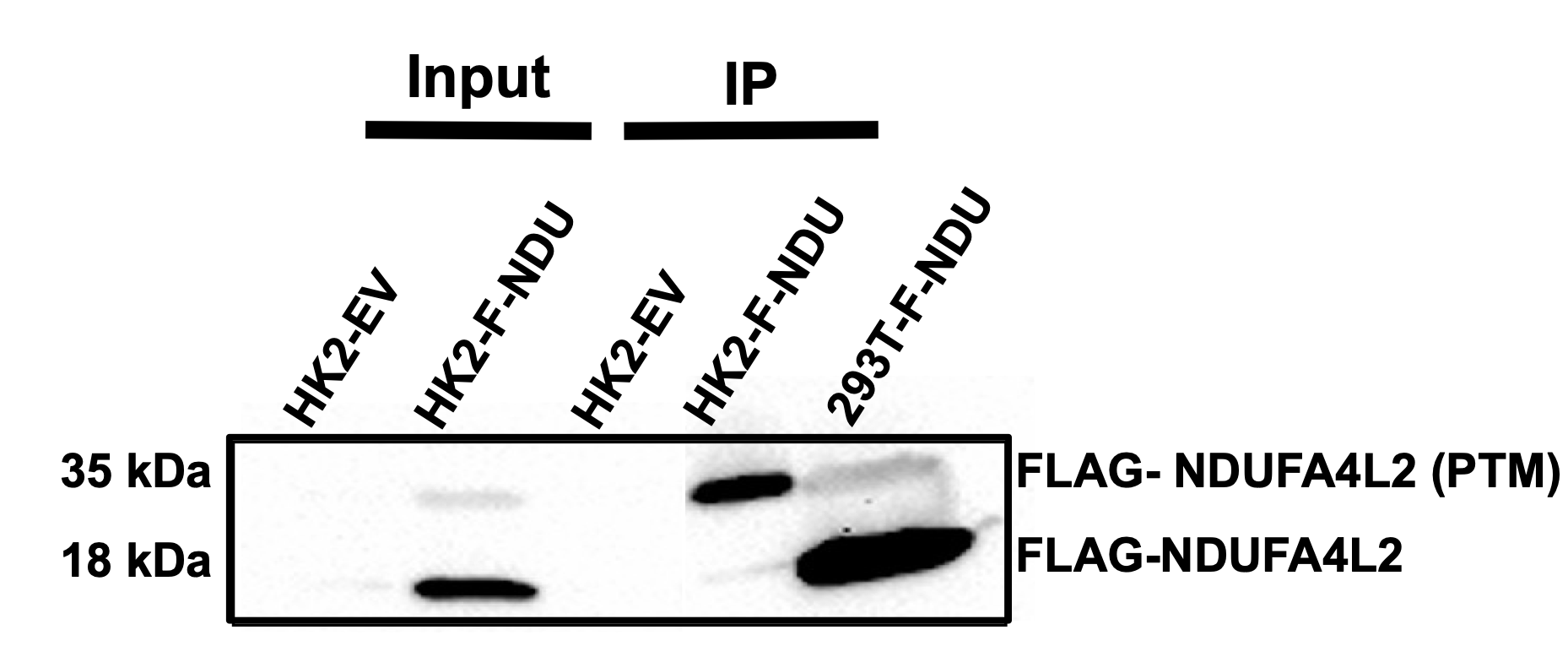

Supplement: Supplemental Material [file KCBT_A_2170669_SM7264.zip › Suppl Figure 1B.tiff]

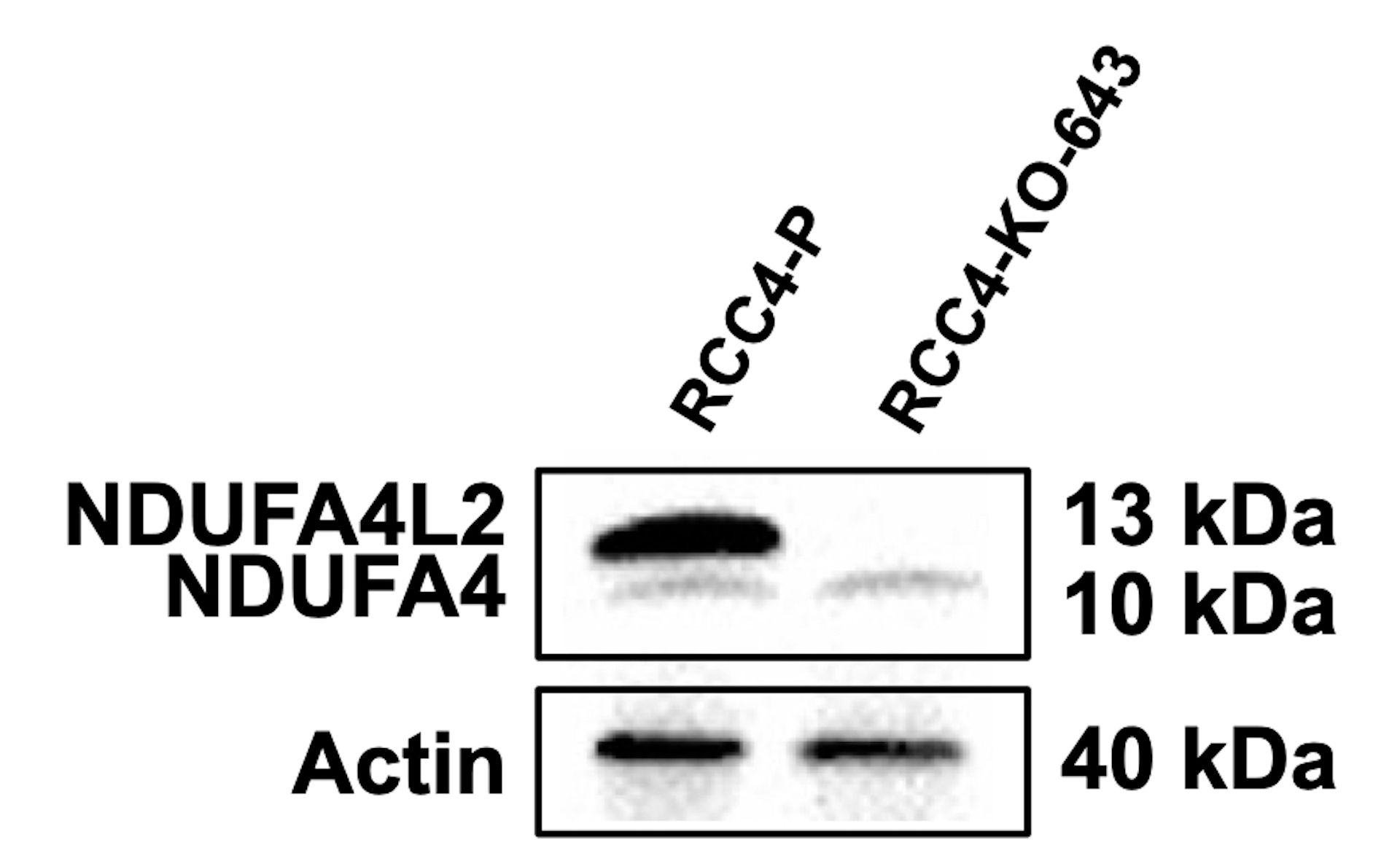

Supplement: Supplemental Material [file KCBT_A_2170669_SM7264.zip › Suppl Figure 1C.tiff]

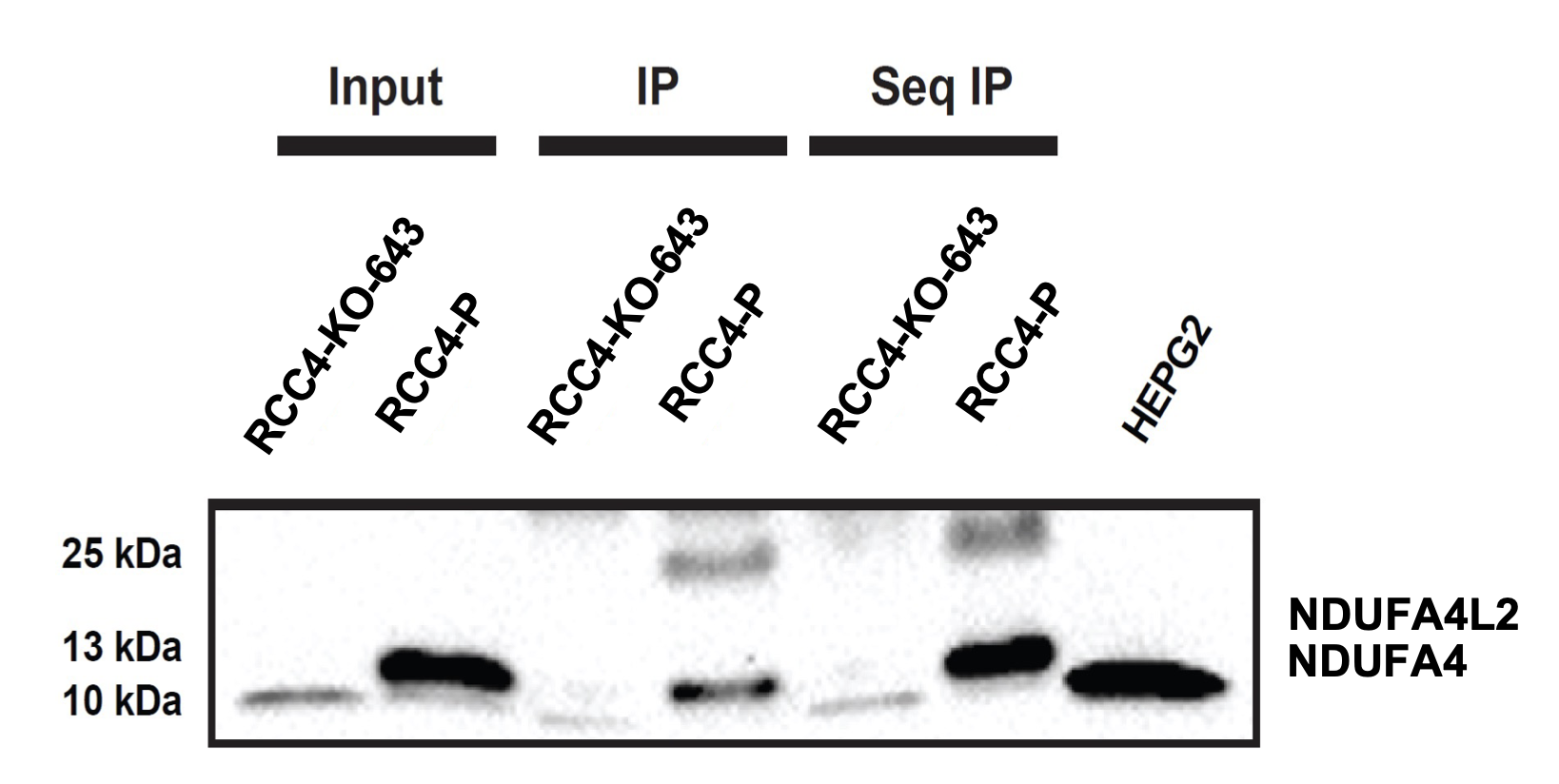

Supplement: Supplemental Material [file KCBT_A_2170669_SM7264.zip › Suppl Figure 1D.tiff]

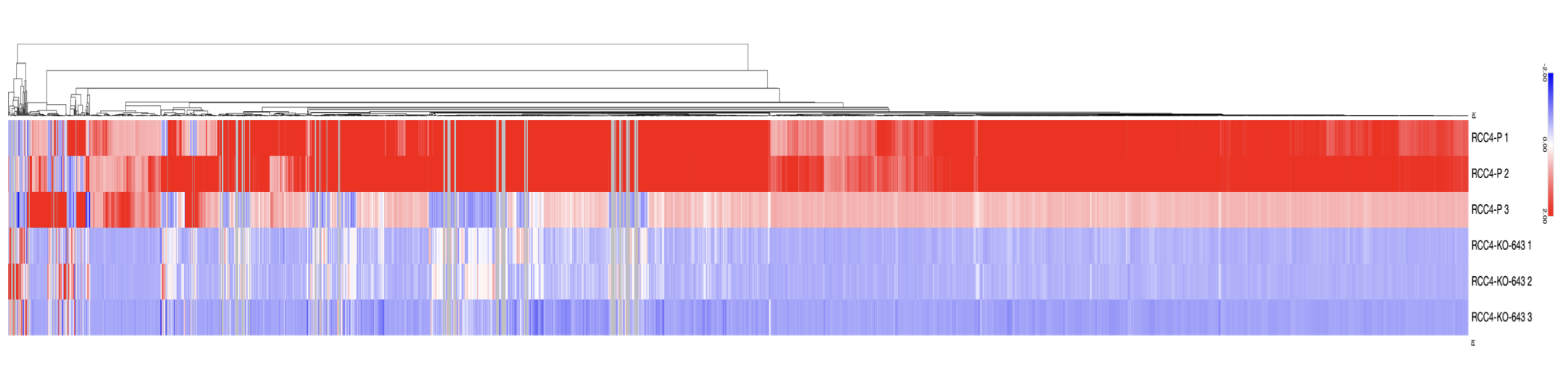

Supplement: Supplemental Material [file KCBT_A_2170669_SM7264.zip › Suppl Figure 2A.tiff]

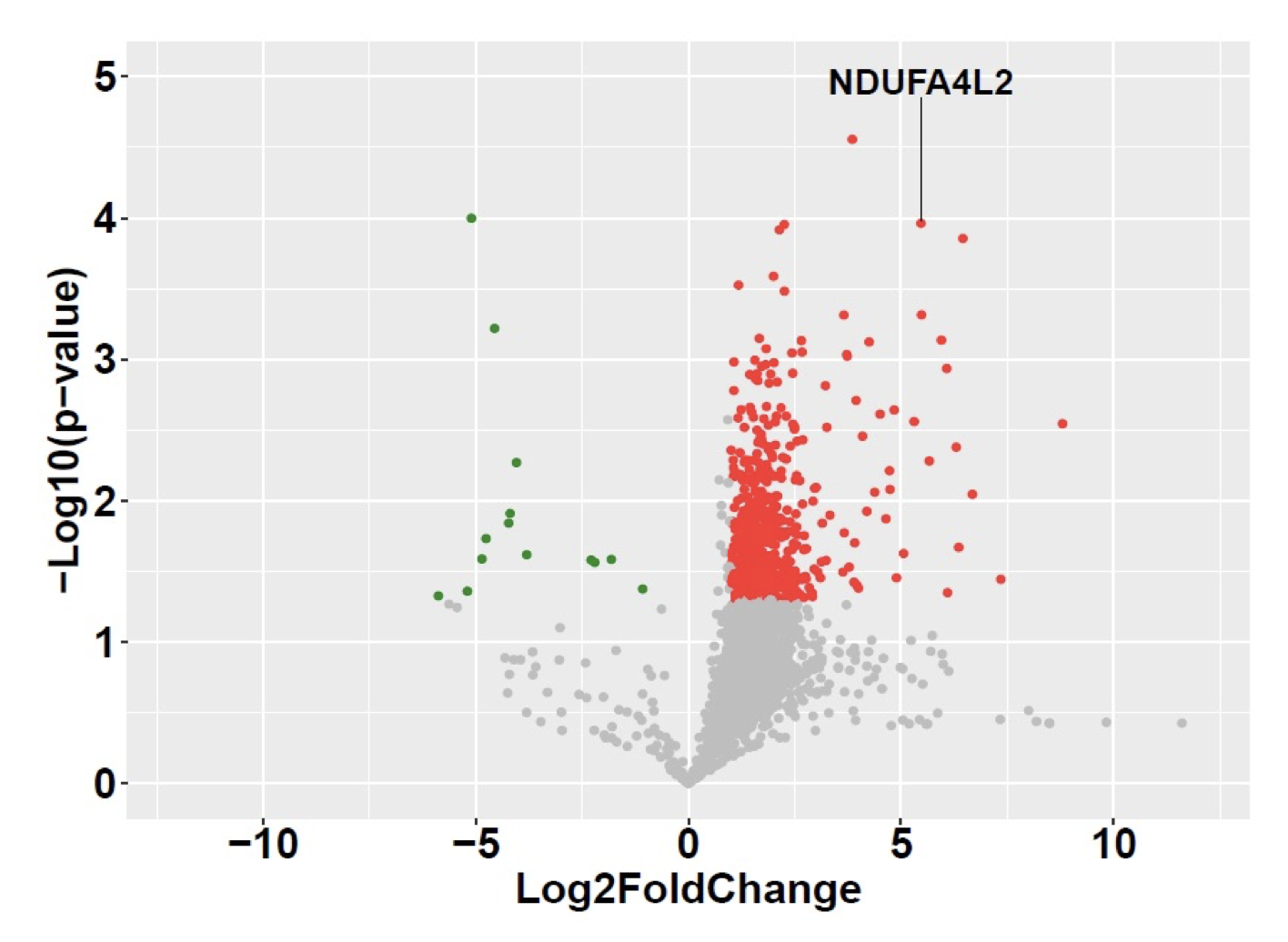

Supplement: Supplemental Material [file KCBT_A_2170669_SM7264.zip › Suppl Figure 2B.tiff]
